# The lowering of the intraocular pressure and the retinal venous pressure by cyclophotocoagulation

**DOI:** 10.1186/s12886-026-04639-6

**Published:** 2026-01-29

**Authors:** Stephan Kremmer, Lara Youssef, Roxana Manoiu, Gerasimos Anastassiou, Michael Selbach, Sascha Klee, Dietmar Link, Richard Stodtmeister

**Affiliations:** 1 Augenzentrum Gelsenkirchen, Ebertstraße 20, 45879 Gelsenkirchen, Germany; 2 Institute for Biomedical Engineering and Informatics, Gustav-Kirchhoff-Straße 2, Technische Universität Ilmenau, 98693 Ilmenau, Germany; 3 Department of General Health Studies, Karl Landsteiner University of Health Sciences, Dr.-Karl-Dorrek-Straße 30, Krems, 3500 Austria; 4 Private Practice, Fichtenstr. 47, 66976 Rodalben, Germany

**Keywords:** Cyclophotocoagulation, Retina, Venous pressure, Intraocular pressure, Blood flow

## Abstract

**Purpose:**

To measure whether cyclophotocoagulation (CPC) can reduce not only intraocular pressure (IOP) but also retinal venous pressure (RVP) in glaucoma patients.

**Patients and methods:**

This retrospective study included 36 eyes of 23 patients with primary open angle glaucoma in whom RVP was measured before and after CPC. In 13 patients both eyes were included in the study and in 10 patients only one eye. Instruments: Applanation tonometry. Measurement of RVP: Slow inflation of a pad lateral to the cornea under observation of the retinal veins (IOPstim, IMEDOS Health GmbH, Jena, Germany). When a venous pulse appears, measurement of the IOP. CPC by IRIS Medical OcuLight SLx diode laser (Iridex Corporation, California, USA). Fifteen spots applied at the inferior 270 degrees.

**Results:**

Pressures are measured in mmHg (median (Q1; Q3); before surgery: IOP = 19.0 (18.0;21.5); RVP = 25.5(23.0;28.5) (*p* < 0.0001) (Wilcoxon test, *N* = 23: averaging of measured values for binocularity). At follow-up 14.3 (11.9;16.1) weeks later: IOP 14.0 (13.0;17.0); RVP = 17.7 (16.0;20.0) (*p* < 0.0001). The median IOP decreased by -5.0 (-7.5; -2.0), the median RVP by -7.8 (-11.0; -4.7) (*P* < 0.01). Both the IOP and the RVP were significantly smaller in the median. The correlation between the magnitude of the pressure-drop in RVP and IOP was low (R^2^ = 0.21).

**Conclusion:**

Both IOP and RVP are significantly reduced by CPC, RVP is more reduced than IOP. Apart from the direct physical effect of IOP lowering on retinal vessels this behaviour may also be caused by a relaxation of the lamina cribrosa with less stress on passing structures like nerve fibers but also on blood vessels.

## Introduction

The main treatment for primary open-angle glaucoma is to reduce the intraocular pressure (IOP) which to this day is assumed to be equal to the *intraocular* venous pressure [1]. This reduction of intraocular venous pressure increases the gradient of blood pressure in the eye and thus improves blood flow. However, during the last 30 years numerous articles have been published showing that the *retinal* venous pressure (RVP) may be higher than the IOP [2–15]. In such cases, the blood pressure gradient in the *retina* is determined by the retinal venous pressure and no longer by the IOP. For the choroid, the previous assumption still applies. The effect of lowering the IOP on the RVP is unclear [16]. This study should provide new insights into this problem.

We investigated whether laser treatment (transscleral cyclophotocoagulation, CPC) [17–19] may not only reduce intraocular pressure but also retinal venous pressure and thus improve the perfusion of the retina, including the optic nerve fibers.

## Methods

This retrospective study included 36 eyes of 23 patients in whom RVP was measured before and after CPC. In 13 patients both eyes were included in the study and in 10 patients only one eye. The study was conducted in accordance with the Declaration of Helsinki and was approved by the ethics committee of the University of Duisburg-Essen Vote No. 24-12282-BO.

The inclusion criteria were as follows: diagnosis: primary open angle glaucoma, IOP without therapy > 21 mmHg, glaucomatous damage of the optic disc, and/or nerve fibers and/or visual field [20].

The transscleral cyclophotocoagulation (CPC) was performed because of insufficient IOP regulation despite medication, eye solution intolerance, or compliance problems.

Exclusion criteria: Lack of fixation. Narrow chamber angle. Spontaneous pulsation of the central vein or of one of its major branches on or near the optic disc. (In the following, only the central retinal vein is mentioned). All eye surgeries except uncomplicated cataract surgery performed six months previously. All eye diseases except glaucoma.

Instruments used: Measurement of IOP: applanation tonometry. RVP was determined with the IOPstim method (IMEDOS Health GmbH, Jena, Germany) [21–23] using an applanation tonometer. This method is described in detail in the cited articles, which is why it is only outlined here: A half-balloon holding 8 mm in diameter is placed on the ocular surface laterally to the limbus in a depressurized state and fixed to a headband. While inspecting the optic disc and its immediate surroundings, the half-balloon is filled with air using a motorized pump until pulsation of the central vein can be seen. At this point, the inflation of the half-balloon is stopped. Immediately afterwards, the IOP is measured. This pressure corresponds to the RVP.

Examination procedure: measurement of IOP, installation of 5 mg/ml tropicamid ophthalmic solution (Mydrum, Dr. G. Mann, Berlin, Germany), measurement of systemic blood pressure. After mydriasis, the IOP was measured. RVP was measured three times, in the quickest possible sequence. The mean value was calculated from these three measurements, which was included in the evaluation as RVP.

Our patients were treated by transscleral cyclophotocoagulation using an IRIS Medical OcuLight SLx diode laser (Iridex Corporation, California, USA). During each procedure, 15 spots were applied at the inferior 270 degrees of the eye with the tip of the laser device positioned at the limbus.

The maximum power output for each spot was 1800–2000 mW, the duration was 2000 msec. As widely recommended, the 3- and 9-o’clock positions were avoided [17, 24].

After CPC, local therapy was continued, supplemented by dexamethasone 1 mg/ml eye solution five times daily (Dexa POS ophthalmic solution, Ursapharm GmbH, Saarbrücken, Germany). At the follow-up examination after CPC, the same diagnostic examinations were performed as preoperatively. The clinical data were taken from the medical records.

The working hypothesis was that the RVP would be reduced by CPC. The null hypothesis was: the RVP is not reduced by CPC. In order to avoid a misjudgement of the probability of error in the confirmative statistics, the mean value was calculated from the RVP values of the left and right eye. This mean value was included in the calculation for this patient. The distribution of the data obtained was assessed using normal distribution plots. Due to the small number of cases (23 patients) from a biometric point of view, the Wilcoxon test was used one-sidedly. The other parameters were tested two-sidedly using the same method for orientation purposes.

## Results

The demographic data of the patients are listed in Table 1.

**Table 1:** Description of the study patients

Description of the patients	
Number	23
Male/female	6/17
Age, years	72(65;81)
BP, systolic, mmHg	130(123;145)
BP, diastolic, mmHg	79(75;85)
MAP; mmHg	95(92;104)
Coronary heart disease	4
Arterial hypertension	15
Arterial hypotension	1
Migraine	2
Hyperlipidemia	9
Apnea syndrome	3
Diabetes mellitus	5
ACEI	10
Systemic ß-blocker	7
Calcium channel blocker	5

Metric parameters: Median (Q1;Q3). BP: Systemic blood pressure; MAP: Mean arterial blood pressure; ACEI: Angiotensin-converting enzyme inhibitor

The 36 operated eyes are described with the data in Table 2.

**Table 2:** Description of the study eyes

*Description of the study eyes*	
Number	36
Duration of the disease, years	3.6(1.7;5.5)
Visual acuity, ccs	0.63(0.50;0.80)
Spherical equivalent, dptr	-0.13(-1.5;0.75)
Central corneal thickness, µm	528(511;552)
*Topical medications*	
Alpha agonists	13
ß-blockers	30
CAH inhibitors	26
Prostaglandin analogs	28
*Earlier laser surgery*	
ALT or SLT	3

**Fig. 1:**
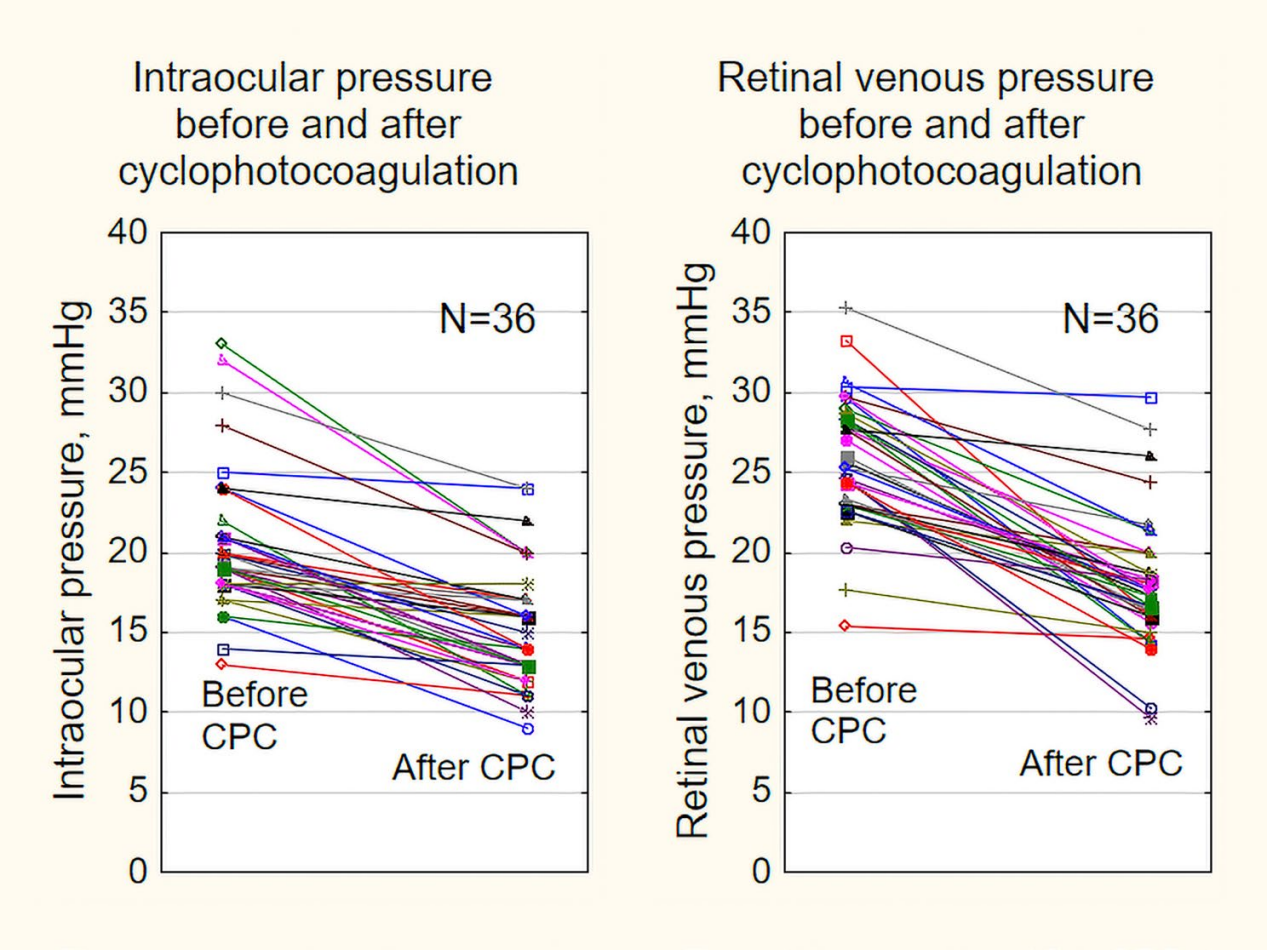
Line diagrams of the intraocular pressure (left) and the retinal venous pressure before and after cyclophotocoagulation

The raw data of IOP and RVP before and after CPC of the 36 eyes are shown as line graphs in Fig. 1.

**Table 3:** Intraocular pressure and retinal venous pressure before and after cyclophotocoagulation given as median (Q1;Q3), p calculated by Wilcoxon-test

Results in 36 eyes before and after CPC Follow up visit after 14.3(11.9;16.1) weeks				
	Intraocular pressure	α	RVP	α
Before CPC	19.0(18.0;21.5)	*P* < 0.0001	25.5(23.0;28.5)	*P* < 0.0001
After CPC	14.0(13.0;17.0)		17.7(16.0;20.0)	

The distribution of RVP values after CPC was no longer compatible with a normal distribution. Therefore, nonparametric methods were used in the evaluation. The median values of these pressures and the result of the statistical test with the Wilcoxon test are shown in Table 3. The differences in IOP values *after CPC* minus IOP values *before CPC* were calculated, as were the differences in RVP. The median IOP decreased by -5.0 (-7.5; -2.0), the RVP decreased by -7.8 (-11.0; -4.7) (*P* < 0.01).

In all 36 eyes, the central vein did not pulsate before CPC. At follow-up, the central vein pulsated in three eyes, so the RVP was assumed to be equal to the spontaneously present IOP [1] and noted as the result.

The RVP, which was included in the statistics, was the mean value of three individual values recorded within 20–30 s. The range of the individual values before CPC was a median of 1.0 (1.0; 2.0) mmHg and after CPC a median of 1.0 (0.5; 1.0.) mmHg. The maximum range of the individual values before and after CPC was 4.0 mmHg. Before CPC, the range was higher than 2.5 mmHg in five eyes and after CPC this was the case in three eyes.

**Fig. 2:**
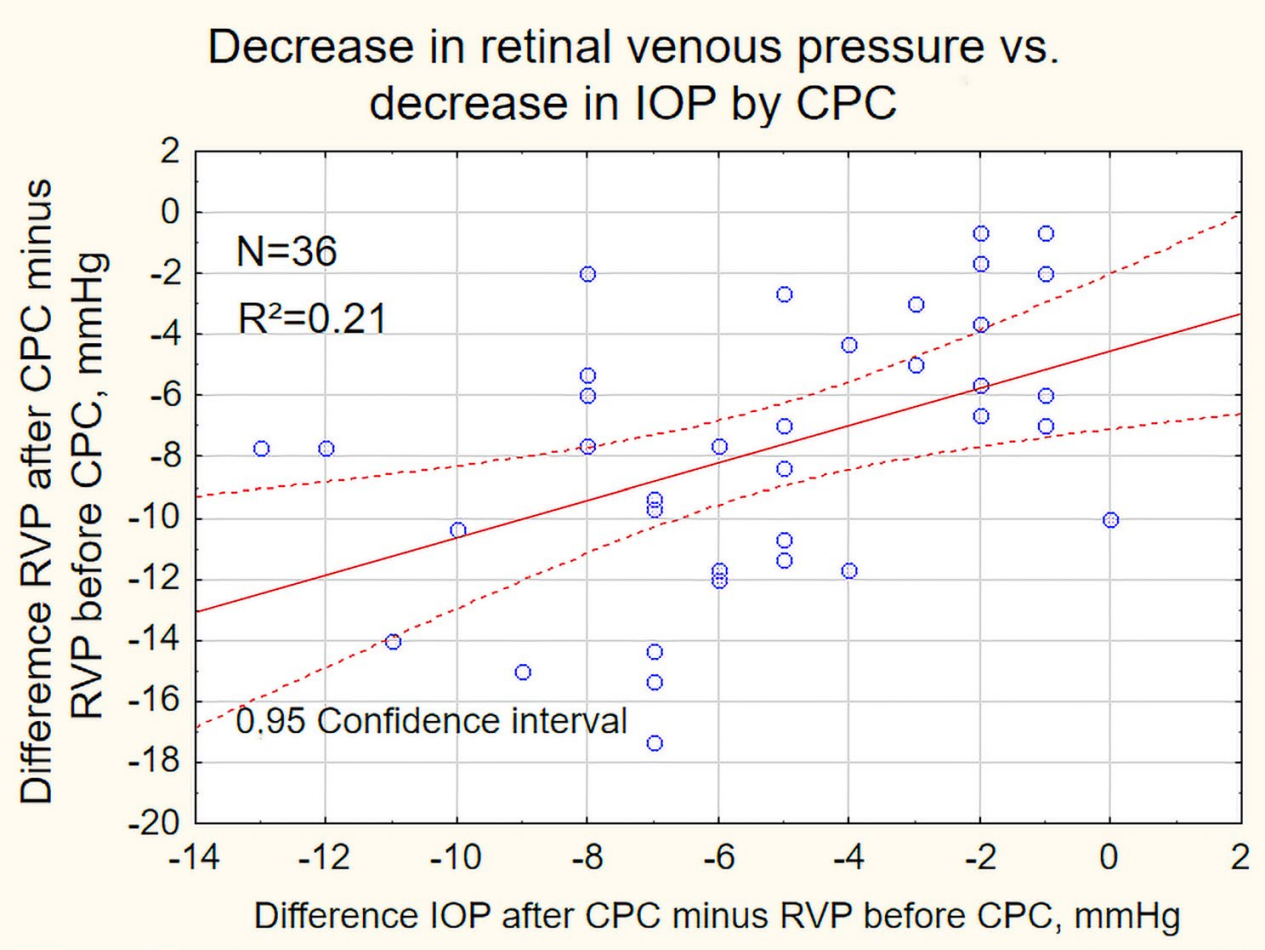
Pressure drop of retinal venous pressure (ordinate) plotted versus pressure drop of the intraocular pressure (abscissa)

The scatter plot in Fig. 2 shows a weak correlation (R²=0.21) between the decrease in RVP and the decrease in IOP.

## Discussion

In Table 3, the values for intraocular pressure and retinal venous pressure before and after CPC are shown as median (Q1;Q3). The intraocular pressure decreased by 5.0 mmHg and the retinal venous pressure by 7.8 mmHg. For a better comparison with earlier results [25], we have calculated mean ± standard error from the values we measured. In this procedure, the initial IOP of 20.5(± 0.75) mmHg was reduced to 15.5(± 0.62) mmHg, i.e. by 5.3 mmHg. The retinal venous pressure decreased from 25.9 ± 0.67 mmHg to 18.1 ± 0.70 mmHg, i.e. by 7.8 mmHg.

In an earlier study from our clinic [25] in 26 patients with primary open angle glaucoma the IOP decreased by the identical CPC procedure from 21.1(± 0.7) mmHg (mean ± standard error) to 16.7 (± 0.6) mmHg, i.e. by 4.4 mmHg. The *median* pressure drop of the IOP was 5.0 mmHg. Because of the relatively small number of cases in both studies, these differences should not be overemphasized.

In the patients of Morgan et al. [16] there were also differences in the drop of IOP and RVP before and after therapy. In their *glaucoma patients*, the drop in IOP was 1.3 mmHg and the drop in RVP was 1 mmHg. Furthermore, in their subjects with *suspected* glaucoma the drop in IOP was 9.3 mmHg and the drop in RVP was 11 mmHg. These authors examined their patients after an average interval of 13.0 months using different surgical methods, including trabeculectomy in five patients. Due to the major differences in the study designs, the results of the two studies will not be compared in detail. However, what the results have in common is that a change in RVP may be possible and may be observed in connection with a change in the same direction as in IOP. As the therapy in this study was not changed apart from the administration of dexamethasone eye drops after CPC, it may be assumed that the reduction in RVP may be solely due to the physical effect of the IOP change. Interestingly, in our study, the effect of CPC on RVP was higher than the achieved IOP reduction alone.

The distribution of the IOP and RVP measurement data was assessed using normal distribution plots. The results of the RVP after CPC were not clearly compatible with a normal distribution. Therefore, the results of IOP and RVP were analyzed using non-parametric methods.

In primates, it has been described that increased intraocular pressure may trigger remodeling of the extracellular matrix of the lamina cribrosa [26, 27]. It is conceivable that this constricts the lumen of the central retinal vessels and consequently may increase the resistance and thus the RVP whereas a relaxation of the lamina cribrosa may cause less stress on passing structures like nerve fibers, but also blood vessels. According to the law of Hagen-Poiseuille, even a small increase in vessel radius may have a major impact in lowering blood flow resistance and improving ocular blood flow [28].

Additionally, a possible cause of increased RVP may thought to be that endothelin may diffuse from the central retinal artery to the central retinal vein, where these two vessels run in close proximity in the optic nerve [29]. This may cause a constriction of the vein. A further reduction in venous resistance may therefore be expected from orally administered vasodilating drugs [13, 30–32]. Initial results in the literature are promising.

According to Gaehtgens’ formula [33], this outflow resistance may be one of the three factors that determine the total blood flow Q of a vascular district:

$$\:Q=\frac{{P}_{c}-{P}_{v}}{{R}_{v}}$$

The resistance R_v,_ (the denominator of the fraction) cannot be measured clinically. The pressure difference between IOP and intracranial pressure may be assigned a role in the development of this resistance. Xie et al. [34] suggest estimating this pressure difference using the MRI-assisted orbital subarachnoid space width, taking into account the body mass index and mean arterial pressure.

The capillary pressure P_c_ cannot be measured clinically either. It may be inferred on the basis of the systemic blood pressure, whereby the measuring points are located at different distances outside the eye depending on the formula selected [35]. When measuring blood pressure on the *upper arm*, the point of measurement is located in the subclavian artery [36] and in *ophthalmodynamometry* it is located in the ophthalmic artery [36]. According to animal experiments, it may be assumed that the pressure in the retinal artery when passing through the lamina cribrosa, is in the order of 100 mmHg. Due to the branching of the retinal vessels and due to autoregulation [37], the blood pressure in the capillaries may be estimated 40 mmHg [33]. Between the clinically discernable points of measurement in blood pressure measurement and the capillaries [36, 38], there are many unknowns that may influence the blood flow. This may be the reason why the capillary pressures calculated according to current formulas may deviate significantly from the actual values.

RVP corresponds to the P_v_ in the formula and is the *only parameter that is clinically directly measurable* with ophthalmodynamometry. In Fig. 2, the reduction in RVP is plotted against the reduction in IOP. The coefficient of determination of 0.21 indicates a very weak correlation, which means that clinically inaccurate values may be expected if RVP is only estimated on the basis of IOP.

Before CPC, the central retinal vein did not pulsate in any of the eyes, but there was pulsation in three eyes after CPC. This behavior could be explained by the model of a Starling resistor [39]. This consists of a compressible tube passing through a vessel with rigid walls [39]. If the pressure in this vessel is higher than the pressure in the tube, the tube shows pulsation at the exit from the vessel. However, if the pressure in the compressible tube is higher than the pressure in the vessel, no pulsation can be seen. In the Starling resistor model, the compressible tube corresponds to the central vein. The occurrence of pulsation in these three patients may therefore be interpreted as a lowering of the RVP. This model might also explain the more frequent absence of pulsation in glaucoma patients as observed by Seo, Kim, Weinreb et al. [40] and Pinto et al. [41]. These authors concluded a causal relationship between pulsation behavior and glaucoma damage simply because of the different frequency of pulsation in healthy and diseased subjects.

Based on these correlations, it seems possible, as demanded by Yang and Weinreb [42], to no longer use the term “normal tension” in glaucoma diagnostics by replacing the IOP with the RVP as a benchmark.

### Limitations

The number of patients examined can be described as small at 23. The results are consistent with those described in the few studies in the field of RVP to date. They may used to establish more far-reaching hypotheses that may then be tested on larger patient cohorts.

The measurement of RVP is a subjective method that is currently not widely used and the accuracy of which may be viewed critically. We have tried to counter these doubts by measuring the RVP three times within 30–60 s and then calculating the mean value from these values. We regarded the range of these three measurements as a quality criterion. Based on the measurement setup, the examiner is blinded to the result, as he observes the fundus with both eyes through the oculars of a slit lamp. Before and after CPC, the median range of these three measured values was 1.0 mmHg. The range was only higher than 2.5 mmHg in five eyes before CPC, and after CPC only in three eyes. In three quarters of the eyes, the range was 2.0 mmHg or less before CPC, and after CPC the range was 1.0 mmHg or less in the same proportion of patients.

Inherent to subjective methods is the possibility that different examiners use different criteria when assessing the threshold characteristic. Since it was the same investigator who examined the patients before and after CPC in this study, this possibility of error may be considered small.

## Conclusion

After CPC, the RVP decreases significantly more than the IOP. This effect may be attributed solely to the physical effect of the intraocular pressure, as the therapy was not changed after CPC with the exception of dexamethasone eye drops.

According to the results presented here, one should not infer the level of reduction in RVP from the reduction in intraocular pressure alone, as the quantitative association is only very loose. It therefore seems appropriate to measure the RVP if the central vein does not pulsate spontaneously.

Future studies need to show whether lowering RVP with oral medication by pentaerythrityltetranitrat [30], l-methylfolat [31], tadalafil [32] or nifedipin [13] may be successful when retinal venous pressure remains higher than intraocular pressure after CPC or surgery. Initial results are promising.

## Data Availability

The data used to support the findings of this study are included in the article and are available on request from the corresponding author.
